# Coverage-Guaranteed Sensor Node Deployment Strategies for Wireless Sensor Networks

**DOI:** 10.3390/s100302064

**Published:** 2010-03-15

**Authors:** Gaojuan Fan, Ruchuan Wang, Haiping Huang, Lijuan Sun, Chao Sha

**Affiliations:** College of Computer, Nanjing University of Post and Telecommunications, 210003, Nanjing, China; E-Mails: wangrc@njupt.edu.cn (R.W.); hhp@njupt.edu.cn (H.H.); sunlj@njupt.edu.cn (L.S.); shachao_2003@163.com (C.S.)

**Keywords:** wireless sensor networks, coverage, desired deployment quality, deployment strategies

## Abstract

Deployment quality and cost are two conflicting aspects in wireless sensor networks. Random deployment, where the monitored field is covered by randomly and uniformly deployed sensor nodes, is an appropriate approach for large-scale network applications. However, their successful applications depend considerably on the deployment quality that uses the minimum number of sensors to achieve a desired coverage. Currently, the number of sensors required to meet the desired coverage is based on asymptotic analysis, which cannot meet deployment quality due to coverage overestimation in real applications. In this paper, we first investigate the coverage overestimation and address the challenge of designing coverage-guaranteed deployment strategies. To overcome this problem, we propose two deployment strategies, namely, the Expected-area Coverage Deployment (ECD) and BOundary Assistant Deployment (BOAD). The deployment quality of the two strategies is analyzed mathematically. Under the analysis, a lower bound on the number of deployed sensor nodes is given to satisfy the desired deployment quality. We justify the correctness of our analysis through rigorous proof, and validate the effectiveness of the two strategies through extensive simulation experiments. The simulation results show that both strategies alleviate the coverage overestimation significantly. In addition, we also evaluate two proposed strategies in the context of target detection application. The comparison results demonstrate that if the target appears at the boundary of monitored region in a given random deployment, the average intrusion distance of BOAD is considerably shorter than that of ECD with the same desired deployment quality. In contrast, ECD has better performance in terms of the average intrusion distance when the invasion of intruder is from the inside of monitored region.

## 1. Introduction

Wireless sensor networks have many applications, including environment monitoring, intrusion detection and tracking, precision agriculture, *etc.* [1,2]. One of the main tasks of wireless sensor networks is the collective monitoring of a field of interest [3]. Therefore, two questions should be taken into consideration before successful monitoring applications: (1) how many sensor nodes have to be deployed to provide a required surveillance level, and (2) how should the sensors be deployed in the monitored region [4]? Generally, coverage is considered as a measure of the quality of service provided by the sensor network [5,6]. In order to sufficiently monitor the entire field of interest for the sensor network, every point of the monitored field must be covered by at least one sensor. Therefore, sensor deployment strategies play a significant role in determining the appropriate placement of sensor nodes to meet certain coverage requirements [7]. The quintessence of sensor deployment is that it uses the least number of sensor nodes to satisfy specific coverage requirement, or to maximize the sensing coverage quality within a given economic budget.

Determining the required number of sensors to be deployed is a critical decision for wireless sensor networks. The art gallery problem is to determine the minimum number of guards required to cover all points in a gallery [8]. Similar works [9] considered the necessary and sufficient conditions for covering a sensor network with nodes deployed in a grid over a square region. Others have mainly focused on the random deployment strategies, e.g., Hall [10] established approximations or asymptotic bounds for area coverage. Based on this asymptotic analysis, Liu *et al.* [11,12] gave the required density to achieve a desired area coverage *f_a_* (0 < *f_a_* <1), in which sensors were uniformly deployed in a 2D infinite plane with Poisson point processes:

(1) $$\lambda =-\text{ln}(1-f_{a})/\pi r^{2}$$

where *r* is the sensing range of sensors, and λ is the density of network. This means that, given the desired area coverage, the minimum number of sensor nodes can be determined by Equation (1). According to asymptotic results, the coverage problem formulation varies to reflect the different objectives and applications: coverage verification [13–16], node scheduling for energy conservation [17–20], and intrusion detection applications [21].

However, these researches assume that the sensors are deployed on an infinite region, rather than boundary region which is more relevant to real application scenarios. Therefore, this asymptotic analysis fails to guarantee the required network coverage in real world applications due to boundary effects [5,22], in which sensors near the border of the monitored region cover less area than sensors placed inside. Actually, the underling analysis results in coverage overestimation, in which the density required by Equation (1) cannot achieve the desired area coverage.

Motivated by the coverage overestimation associated with uniform random deployment, in this paper, we propose two novel random deployment strategies for sensor nodes with coverage-oriented requirement, namely, Expected-area Coverage Deployment (ECD) and BOundary Assistant Deployment (BOAD).

The main contributions of this paper are the following: (1) we formulate the coverage overestimation for random deployed sensor networks; (2) we propose two deployment strategies to solve the coverage overestimation problem and analyze the lower bound on the number of deployed sensor nodes for each strategy to fulfill the deployment quality; (3) we carry out performance evaluation and demonstrate that the proposed deployment strategies can effectively alleviate the coverage overestimation and achieve user-specified desired deployment quality; (4) we apply two strategies to the application of intrusion detection to investigate the tradeoff between the deployed quality and average intrusion distance under two intrusion scenarios.

The remainder of this paper is organized as follows. In Section 2 related works are outlined. Section 3 describes the system models and problems. Random deployment strategies are proposed and the impact of deployment on coverage is discussed in Section 4. In Section 5, the performance of the deployment strategies is evaluated and compared. In Section 6, we discuss some practical issues, such as the extensibility of our work. Finally, we draw the conclusion, and point out the future work in Section 7.

## 2. Related Work

Sensor deployment is a critical issue since it reflects the cost and the surveillance capability of a wireless sensor network. Therefore, a great deal of research has studied the deployment problem related to sensing coverage. The problem of sensor placement in a monitored field has been investigated in depth in [23]. The required number of sensor nodes and their places were determined in [9] to provide a coverage threshold that defines the confidence level of the deployment. Coskun *et al.* [24] used the hexagonal grid positioning method to achieve maximum cumulative connected coverage. However, the problem formulations and their solutions in these works depend on the exact positions. As studied in [25], it is unrealistic to expect all sensors to be placed exactly on the grid vertices due to placement errors.

Random deployment refers to the situation in which sensor nodes are uniformly and independently distributed across the monitored field. In [10], Hall studied how many nodes with fixed coverage radius are needed so that every point of a unit square region is covered by randomly placed sensor nodes. The research in [11,12] determined the densities of sensor nodes that achieve a desired area coverage based on Hall’s asymptotic analysis. They defined the area coverage as the fraction of the geographical area and determined the minimum number of sensors to be deployed in the infinite plane using homogeneous Poisson point processes.

Recently, most researches have extended the above analysis to coverage verification or coverage analysis. Tsai [13] addressed the sensing coverage for a randomly distributed sensor network in the shadowed environments. The basic observation is that the shadowing effects make impact on the sensing coverage, and the sensing coverage severely degrades with the increase of standard deviation of the shadowing effects. An algorithm was proposed in [14] to achieve tradeoff between the cost of deployment and the percentage of area covered under random deployment. In [15], the issue of estimating the number of sensors required to achieve complete coverage for a desired region was studied. The coverage holes were introduced in [16] as a metric to evaluate the performance of deployment strategies with the presence of failures and placement errors in sensor networks.

In addition, the theory of asymptotic analysis also has a great impact on coverage based node scheduling, active node selecting and applications such as intrusion detection. Given the assumption that the nodes are densely deployed, the research in [17] and [18] organized the sensor nodes into disjoint node sets by working alternately to extend network lifetime. The number of nodes in one set was selected according to the coverage requirement. Moreover, the research in [19] and [20] considered energy conservation by taking coverage and connectivity into consideration. On the other hand, Wang in [21] deployed sensors randomly for intrusion detection application and characterized the deployment parameters such as node density and sensing range in terms of the desirable detection probability.

All these random deployment strategies and their corresponding applications focused on the analysis results that the number of sensor nodes deployed or selected can achieve desired coverage requirement. Unfortunately, as shown in the empirical study in [5,22], these analytical expressions may induce coverage overestimation whereby the number of sensors deployed or active nodes selected fails to meet the required coverage quality. In other words, the minimum number of sensors analyzed is smaller than the practical number of sensors to achieve desired network coverage due to the boundary effect. The results in [13] indicated that the numerical results more optimistic outcomes than those obtained by simulation. In practice, their results could be more convincing if boundary effects are considered.

In this paper, we intend to study random sensor node deployment strategies concerning with coverage guaranteed. The goal of our design is to meet the coverage requirement of a sensor network by using a minimum number of sensor nodes randomly deployed in a certain area.

## 3. Network Model and Problem Description

In this section, we describe the network model and give some definitions to simplify the analytical process in Section 4.

### 3.1. Network Model

We consider the sensor nodes randomly and uniformly deployed in the monitored field. Assume that the sensing area is the disk of radius centered at the sensor with sensing radius *r*. In this model, each sensor node can only sense the environment and detect events within its sensing range. A point is said to be covered by sensor if and only if it falls in the sensing range of at least one sensor, and the monitored region is said to be covered if each point in this region is covered [11].

To facilitate later discussion, we introduce the following definitions.

**Definition 1.** A monitored region Ω. A monitored region is defined as the area monitored by sensor nodes. We consider this area as *l* × *m* rectangular monitored region.

**Definition 2.** Desired deployment quality (DDQ). For a sensor network, the desired coverage region is an area which is covered by deployed sensor nodes, which means the area that can be covered by a sensor network. Therefore, the desired deployment quality of a sensor network is defined as expected proportion of the desired coverage region to the monitored region [26].

(2) $$\frac{\left\|(\cup_{i=1}^{n}S(r_{i}))\cap \Omega \right\|}{\left\|\Omega \right\|}$$

where *n* is the number of deployed sensor nodes in monitored region, *S*(*r_i_*) is the sensing area of sensor *i*, and ||Ω|| is the size of the monitored region Ω.

### 3.2. Problem Description

Given a monitored region Ω, and the sensing area of each sensor *S*(*r_i_*), we need to consider how one should deploy a given number of sensor nodes so that the network coverage can meet the userspecified DDQ.

(3) $$\begin{array}{l} \mathrm{Minimize}\;\;n\\ \mathrm{subject to}\;:\;r_{1}=r_{2}=\ldots =r_{n}=r\\ \;\;\;\;\;\;\;\;\;\;\;\;\;\;\;\;\;\;\frac{\left\|(\cup_{i=1}^{n}S(r_{i}))\cap \Omega \right\|}{\left\|\Omega \right\|}\geq \text{DDQ}\\ \;\;\;\;\;\;\;\;\;\;\;\;\;\;\;\;\;\;\;\;\;\;\;\;\;\;0<S(r_{i})\leq \pi r^{2} \end{array}$$

where DDQ is the desired deployed quality requirement mentioned in definition 2.

The notations used in this paper are listed in Table 1.

## 4. Random Deployment Strategies

In this section, we proposes two novel deployment strategies, namely, Expected-area Coverage Deployment (ECD) and BOundary Assistant Deployment (BOAD), respectively.

### 4.1. Expected-Area Coverage Deployment Strategy

In practical applications, the points near the boundary of monitored region for an area coverage deployment (ACD) strategy have a smaller chance to be covered by the sensor, which would decrease network coverage requirement. Therefore, the analytical expressions of Equation (1) could exhibit the coverage holes in real deployments.

For an arbitrary node *i*, define its distance from the boundary of monitored region as *d*(*i*), and the intersection area of its sensing coverage and the monitor region as *Ef*(*d*(*i*)), which is also the effective coverage area of sensor *i*. Therefore, for the nodes where *d*(*i*) ≥ *r*, the effective coverage area is equal to the sensing coverage, *i.e.*, *Ef*(*d*(*i*)) = π*r*^2^. For the nodes where 0 ≤ *d*(*i*) < *r*, it holds that *Ef*(*d*(*i*)) < π*r*^2^. Therefore, we wish to determine the expected area covered by *n* deployed sensor nodes in the monitored region.

**Figure 1.**
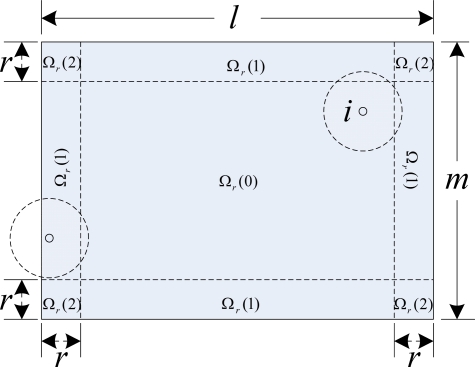
A monitored region and its sub-region.

For *r* > 0 and a monitored region Ω, let us partition Ω into three types of sub-regions Ω*_r_*(0), Ω*_r_*(1), Ω*_r_*(2) as illustrated in Figure 1. And the areas of these three regions are denoted as follows:

(4) $$\{\begin{array}{l} \left\|\Omega_{r}(0)\right\|=\frac{\left(l-2r)(m-2r\right)}{\mathrm{lm}}\\ \left\|\Omega_{r}(1)\right\|=\frac{2r((l-2r)(m-2r))}{\mathrm{lm}}\\ \left\|\Omega_{r}(2)\right\|=\frac{4r^{2}}{\mathrm{lm}} \end{array}$$

We use the variable *V* to represent the area in Ω not be covered by sensor nodes. We have:

(5) $$V=\int_{\Omega }\chi (x)\mathrm{dx}$$

where

(6) $$\chi (x)=\{\begin{array}{ll} 1 & \mathrm{x is not covered}\\ 0 & \mathrm{otherwise} \end{array}$$

Using Fubini’s theorem, we have:

(7) $$E(V)=\int_{\Omega }E\{\chi (x)\}\mathrm{dx}$$

For an arbitrary point x∈Ω, let *A*(*x*) denote a circular area centered at *x* with radius *r*, which means that *A*(*x*) = *πr*^2^. Point *x* is identified to be covered if there is at least one of the deployed sensor *i*, within *A*(*x*). Therefore:

(8) E{χ(x)}=P{x is not covered}

To measure *E*{*χ*(*x*)}, we first measure probability *p_xi_* that a point *x*∈Ω will not be covered by a deployed sensor *i*. Assume that point *x* is selected over Ω as an event. For this event, there are three possible outcomes: (a) *x* lies inside Ω*_r_*(0); (b) *x* lies inside Ω*_r_*(1); (c) *x* lies inside Ω*_r_*(2). These three events are mutually exclusive. Therefore, the probability *p_xi_* that point *x* is not covered by a randomly deployed sensor is given by:

(9) $$p_{\mathrm{xi}}=1-(\int_{\Omega_{r}(0)}\frac{E_{r}\left[0\right]}{\left\|\Omega \right\|}\mathrm{dx}+\int_{\Omega_{r}(1)}\frac{E_{r}\left[1\right]}{\left\|\Omega \right\|}\mathrm{dx}+\int_{\Omega_{r}(2)}\frac{E_{r}\left[2\right]}{\left\|\Omega \right\|}\mathrm{dx})$$

where *E_r_*[*j*] (*j*=0,1,2) is the expected coverage area that an arbitrary sensor is located on the point *x* in sub-region Ω*_r_*(*j*).

For *x*∈Ω*_r_*(0), we have:

(10) $$E_{r}[0]={\pi r}^{2}$$

The computing of *E_r_*[1] and *E_r_*[2] are described in Appendix. Then, we have:

(11) $$E_{r}\left[1\right]=(\pi -\frac{2}{3})r^{2}$$

(12) $$E_{r}\left[2\right]=(\pi -\frac{29}{24})r^{2}$$

Then, Equation (9) can be rewritten as:

(13) $$p_{\mathrm{xi}}=1-\frac{{\pi r}^{2}\mathrm{lm}-\frac{4}{3}r^{3}l-\frac{4}{3}r^{3}m+\frac{1}{2}r^{4}}{{\left(\mathrm{lm}\right)}^{2}}$$

Therefore, when *n* sensor nodes are deployed in Ω, we get:

(14) $$E\{\chi (x)\}=\prod_{i=1}^{n}p_{\mathrm{xi}}={\left(p_{\mathrm{xi}}\right)}^{n}$$

and:

(15) $$\begin{array}{ll} E(V) & =\int_{\Omega }{\left(p_{\mathrm{xi}}\right)}^{n}\mathrm{dx}\\ & =\int_{\Omega }{\left(1-\frac{{\pi r}^{2}\mathrm{lm}-\frac{4}{3}r^{3}l-\frac{4}{3}r^{3}m+\frac{1}{2}r^{4}}{{\left(\mathrm{lm}\right)}^{2}}\right)}^{n}\mathrm{dx}\\ & =\left\|\Omega \right\|{\left(1-\frac{{\pi r}^{2}\mathrm{lm}-\frac{4}{3}r^{3}l-\frac{4}{3}r^{3}m+\frac{1}{2}r^{4}}{{\left(\mathrm{lm}\right)}^{2}}\right)}^{n} \end{array}$$

Based on above analysis, we give the theorem 1.

**Theorem 1.** If *n* sensor nodes with sensing radius *r* are deployed uniformly and randomly in a monitored region Ω, the DDQ by ECD strategy is given by:

$${\text{DDQ}}^{\left(\text{ECD}\right)}=1-{\left(1-\frac{{\pi r}^{2}\mathrm{lm}-\frac{4}{3}r^{3}l-\frac{4}{3}r^{3}m+\frac{1}{2}r^{4}}{{\left(\mathrm{lm}\right)}^{2}}\right)}^{n}$$

**Proof.** Since:

(16) $$\text{DDQ}=1-\frac{E(V)}{\left\|\Omega \right\|}$$

From Equation (15), we have:

(17) $${\text{DDQ}}^{\left(\text{ECD}\right)}=1-\frac{E(V)}{\left\|\Omega \right\|}=1-{\left(1-\frac{{\pi r}^{2}\mathrm{lm}-\frac{4}{3}r^{3}l-\frac{4}{3}r^{3}m+\frac{1}{2}r^{4}}{{\left(\mathrm{lm}\right)}^{2}}\right)}^{n}$$

**Lemma 1.** Assuming that sensor nodes with sensing radius *r* are randomly deployed in *l* × *m* rectangle monitored region. Given the deployment quality requirement *q* < 1, the lower bound number of ECD strategy is:

(18) $$n^{\left(\text{ECD}\right)}\geq \left\lceil \frac{\text{ln}(1-q)}{\text{ln}(1-\frac{{\pi r}^{2}\mathrm{lm}-\frac{4}{3}r^{3}l-\frac{4}{3}r^{3}m+\frac{1}{2}r^{4}}{{\left(\mathrm{lm}\right)}^{2}})}\right\rceil$$

**Lemma 2.** For sensor nodes with sensing range *r* are randomly and uniformly distributed in *l* × *m* rectangular monitored region, the expected coverage area of the sensor is:

(19) $$E\left[r\right]=\frac{{\pi r}^{2}\mathrm{lm}-\frac{4}{3}r^{3}l-\frac{4}{3}r^{3}m+\frac{1}{2}r^{4}}{\mathrm{lm}}$$

**Proof.** From Figure 1 and Equations (10), (11), and (12), we get:

$$\begin{array}{ll} E\left[r\right] & =\left\|\Omega_{r}\;(0)\right\|\times E_{r}\;\left[0\right]+\left\|\Omega_{r}\;(1)\right\|\times E_{r}\;\left[1\right]+\left\|\Omega_{r}\;(2)\right\|\times E_{r}\;\left[2\right]\\ & =\frac{\left(l-2r)(m-2r\right)}{\mathrm{lm}}\times {\pi r}^{2}+\frac{2r((l-2r)+(m-2r))}{\mathrm{lm}}\times (\pi -\frac{2}{3})r^{2}+\frac{4r^{2}}{\mathrm{lm}}\times (\pi -\frac{29}{24})r^{2}\\ & =\frac{{\pi r}^{2}\mathrm{lm}-\frac{4}{3}r^{3}l-\frac{4}{3}r^{3}m+\frac{1}{2}r^{4}}{{\left(\mathrm{lm}\right)}^{2}} \end{array}$$

### 4.2. Boundary Assistant Deployment Strategy

An alternative, and possibly more counter-intuition, approach is deploying sensor nodes outside the monitored region for dealing with the above coverage overestimation. In this section, we propose boundary assistant deployment strategy (BOAD). In contrast to the ECD strategy, sensor nodes are deployed not only in the monitored region but also in a boundary assistant region in BOAD strategy. First of all, we give the definition of boundary assistant region as following.

**Figure 2.**
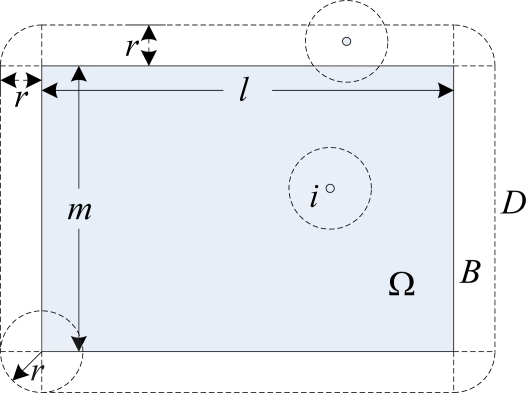
A sensor deployment region.

**Definition 3.** A boundary assistant region *B*, is a peripheral area of monitored region that walks along the boundary of a monitored region at a distance *r* [27]. Like white region shown in Figure 2, we have ||B|| = 2*r*(*l* + *m*) + π*r*^2^.

**Definition 4.** A sensor deployment region *D* is the boundary-assistant region *B* plus monitored region Ω.

From the Figure 2, it is clear that the area of deployment region *D* is:

(20) $$\left\|D\right\|=\mathrm{lm}+2r(l+m)+{\pi r}^{2}$$

Therefore, any point *x* within Ω is considered to be covered if it is inside the sensing range of at least one sensor within *D*. We first measure *p_xi_* that a point *x*∈Ω will not be covered by a deployed sensor *i*. When sensor *i* is not located in *A*(*x*), *i.e.*, *i*∈*D* − *A*(*x*), the point *x* will not be covered. Therefore, the probability that the point is not covered by a randomly deployed sensor is given by:

(21) $$p_{\mathrm{xi}}=\int_{D-A(x)}\rho (x)\mathrm{dx}$$

where *ρ*(*x*) is the probability that deployed sensor *i* is located on the point within *D*. For the uniformly and randomly distributed sensors, we have *ρ*(*x*) = 1/*D*. Hence Equation (21) above can be rewritten as:

(22) $$p_{\mathrm{xi}}=\int_{D-A(x)}\frac{1}{\left\|D\right\|}\mathrm{dx}=1-\frac{A(x)}{\left\|D\right\|}$$

Obviously, for the given number of sensor nodes *n* which should be deployed in Ω, we get:

(23) $$E\{\chi (x)\}=\prod_{i=1}^{n}p_{\mathrm{xi}}={\left(p_{\mathrm{xi}}\right)}^{n}$$

and:

(24) $$E(V)=\int_{D}{\left(p_{\mathrm{xi}}\right)}^{n}\mathrm{dx}=\int_{D}{\left(1-\frac{A(x)}{\left\|D\right\|}\right)}^{n}\mathrm{dx}=\left\|D\right\|{\left(1-\frac{A(x)}{\left\|D\right\|}\right)}^{m}$$

Finally, when sensor nodes are uniformly and randomly deployed within *D*, we state the Theorem 2.

**Theorem 2.** Given *n* sensor nodes with sensing radius *r* are uniformly and randomly deployed within *D*, the DDQ by BOAD strategy is:

(25) $${\text{DDQ}}^{\left(\text{BOAD}\right)}=1-\frac{E(V)}{\left\|D\right\|}=1-{\left[1-\frac{{\pi r}^{2}}{\mathrm{lm}+2r(l+m)+{\pi r}^{2}}\right]}^{n}$$

**Lemma 3.** Given the monitored region Ω and the sensing radius *r*, the lower bound on the number of sensor nodes to meet the deployment requirement *q* < 1 under the BOAD strategy is expressed as:

(26) $$n^{\left(\text{BOAD}\right)}\geq \left\lceil \frac{\text{ln}(l-q)}{\text{ln}(1-\frac{{\pi r}^{2}}{\mathrm{lm}+2r(l+m)+{\pi r}^{2}})}\right\rceil$$

## 5. Performance Evaluation

In this section, the performance of two deployment strategies was evaluated using simulations and compared with the deployment strategy analyzed in [11,12], which we call area coverage deployment (ACD) strategy. Our simulation includes three parts as follows. In the first part of simulation, presented in Section 5.1, the performance of deployment strategies was studied with regard to coverage constraint. The second part of simulation that intrusion detection is used as an example to evaluate the performance. In the third part of simulation, the effects of network parameters on the deployment quality were given.

### 5.1. Performance of Three Deployment Strategies

We first investigate the performance of the three deployment strategies. The simulations are maked on a 100 m × 100 m monitored field with a sensing radius of 15 m, and the desired deployment quality *q* is calculated to ensure that the ratio of coverage in an initial deployment is no less than *q*. We are interested in measuring the performance of the proposed deployment strategies by evaluating the following metrics with desired deployment quality varying from 0.7 to 0.99 with 0.5 intervals. Each value plotted on the figure is the average result of 100 randomly generated topologies.

1. The minimum number of deployed sensor nodes: a measure of deployment cost to achieve the desired deployment quality.
2. Deployment quality achieved and deployment errors: a measure of efficiency for deployment quality.

**Figure 3.**
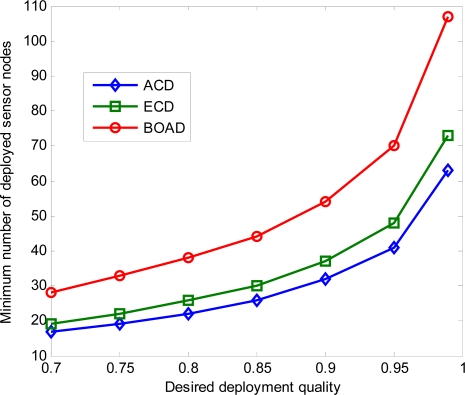
The minimum number of sensors deployed to meet desired deployment quality.

In Figure 3 we study the relationship between the minimum number of deployed sensor nodes and the desired deployment quality. It can be observed that the minimum number of deployed sensors increases with the desired deployment quality increases. Both ECD and BOAD require more sensor nodes to achieve the desired deployment quality than that of deployed sensors in ACD. For example, given the desired deployment quality *q* = 0.90, at least 32 sensor nodes are required to be deployed in ACD. However, at least 37 and 54 sensor nodes are needed, respectively, to attain the same deployment quality in ECD and BOAD strategies. This is consistent with the conclusion drawn in [5]. The reason for this is that ACD considers the sensing areas of deployed sensor nodes are included in the monitored region completely, which cannot achieve the desired deployment quality in the simulation.

Meanwhile, the minimum number of sensor nodes that have to be deployed increases drastically when the desired deployment quality reaches a certain threshold for three strategies. When *q* = 0.95, at least 41, 48, and 70 sensor nodes are needed for ACD, ECD and BOAD, respectively. Moreover, at least 63, 73, and 107 sensors are needed to be deployed with the desired deployment quality *q* = 0.99 in ACD, ECD and BOAD, respectively. Furthermore, there are at least 73, 84, and 123 sensor nodes are to be deployed when the desired deployment quality *q* = 0.999. This implies that, before deployment, we should effectively evaluate the number of sensor nodes to fulfill the required deployment quality. The figure also shows that the desired deployment quality is infinitely close to 1 when the number of sensor nodes is larger than a certain threshold.

**Figure 4.**
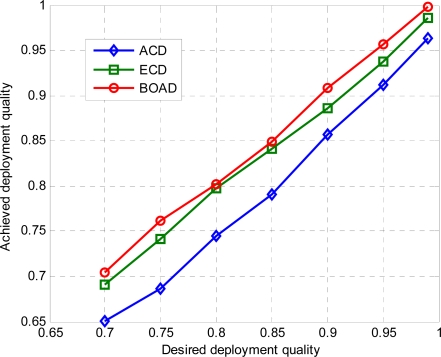
Desired deployment quality and achieved deployment quality.

**Figure 5.**
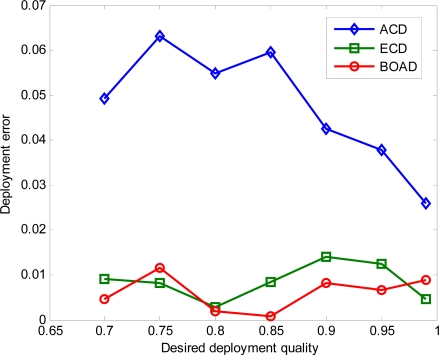
Deployment error.

Figure 4 shows the relationship between desired deployment quality and achieved deployment quality by the number of deployed sensors as demonstrated in Figure 3. It can be seen that the two proposed deployment strategies ECD and BOAD can achieve the real deployment requirement and outperform the ACD on desired deployment quality. That is, the minimum number of deployed sensors in BOAD can sufficiently satisfy the desired deployment quality all the time. On the other hand, ECD meets the desired deployment quality sometimes. Comparably, ACD hardly achieves the requirement because its analysis is based on the scenario that the monitored region is infinite. The deployment errors under three strategies are plotted in Figure 5.

From Figure 5, we can observe that BOAD shows the smallest deployment errors with the help of boundary assistant region. ECD has a slightly larger deployment errors compared to BOAD. However, ACD has the largest deployment error among the three deployment strategies. For example, the deployment discrepancy is 0.0631 for ACD. On the contrary, there are at most 0.0141 discrepancy between simulation results and the analytical findings both in BOAD and ECD. However, BOAD requires significantly more sensor nodes for a given level of desired deployment quality than that of ECD as demonstrated in Figure 3, but offers very small improvement. To efficiently evaluate the performance of deployment strategies, we use intrusion detection as an application example to show the detection performance in Section 5.2.

### 5.2. Detection Application

Intrusion detection in a wireless sensor network can be regarded as a monitoring system for detecting the intruder that is invading the monitored region [21]. In intrusion detection applications, one concern is the speed that the intruder can be detected by the sensor nodes. The intrusion distance is the distance that the intruder travels before it is detected by a network for the first time. Specifically, it is the distance between the point where the intruder enters the monitored region and the point where the intruder gets detected by any sensors. Hence, the intrusion distance is an important metric to evaluate the quality of deployment.

To conduct a convincing performance evaluation and a fair comparison, we use the average intrusion distance as a metric to explore and compare the performance of the three deployment strategies. In the simulations, the sensors with sensing radius *r* = 5, 15 m and the monitored region is 100 m × 100 m. The number of deployed sensor nodes is determined by the desired deployment quality. Each data point in the following figures is the average of 1,000 simulation results.

**Figure 6.**
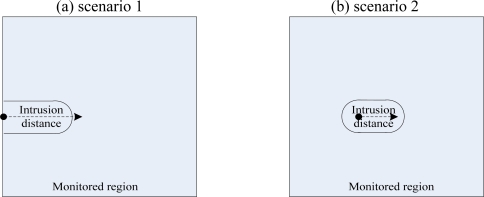
Intrusion scenarios. (a) The intrusion from the boundary of the monitored region.

In the simulations, we assume that the intruder’s physical size can be neglected, and the intruder in the monitored region moves along a straight line at a constant speed. Based on the starting point where the intruder makes its initial intrusion, we conclude two intrusion manners proposed in [28] as the following two scenarios: (a) the intruder can start its intrusion from the boundary of the monitored region (or outside the monitored region) or, (b) the intruder starts at a random point inside the monitored region. Therefore, Figure 6 shows two intrusion scenarios discussed above.

**Figure 7.**
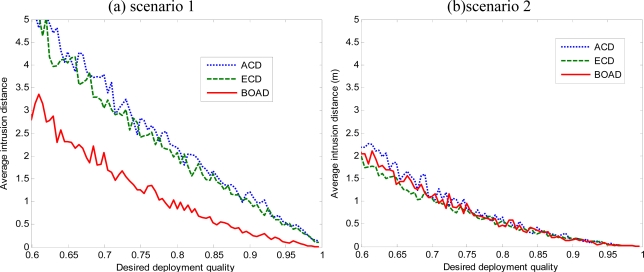
Comparison average intrusion distance of three deployment strategies with sensing radius *r* = 5 m. (a) The intrusion comes from the boundary of the monitored region.

**Figure 8.**
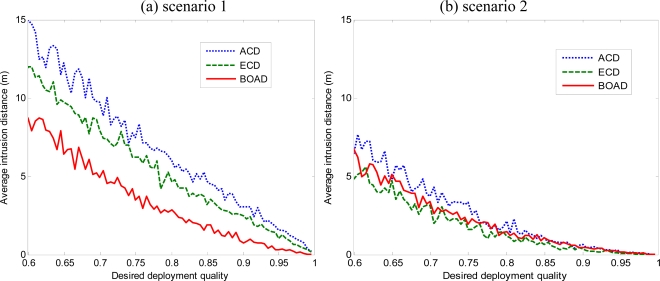
Comparison average intrusion distance of three deployment strategies with sensing radius *r* = 15 m. (a) The intrusion comes from the boundary of the monitored region. (b) The intrusion comes from inside the monitored region.

Figure 7 and Figure 8 present the comparison of average intrusion distance with various values of DDQ in three deployment strategies. The average intrusion distance decreases with the increase of DDQ. For the 100 m × 100 m monitored area, the maximum intrusion distance is $100\;\sqrt{2}m$. Given a fixed DDQ, the average intrusion distance of ACD is the largest one compared to the two others. Because of coverage overestimation, the number of sensor deployment fails to achieve the intrusion applications. We also observe that, with high desired deployment quality, the intruder can be immediately detected once it approaches the monitored area. However, with low desired deployment quality, it is able to detect a moving intruder within a certain intrusion distance.

From Figures 7 and 8, it can be seen that the proposed strategies present a clear performance advantage over the ACD, especially when *r* is larger. Moreover, BOAD strategy performs uniformly better than ECD in terms of the average intrusion distance when the intruder makes intrusion at the boundary of monitored region (see Figures 7a and 8a). This is because BOAD uses the boundary assistant region deployment and the nodes deployed have more chance to cover the point at the boundary of monitored region by the BOAD strategy. On the other hand, ECD has the best performance in terms of the average intrusion distance when the intruder comes from inside the monitored region. This is because when the sensor nodes deployment in the monitored region, the detection probability is higher in the inside of the monitored region than at the border.

It is worth noting that the average intrusion distance is longer when *r* = 15 m than *r* = 5 m given a fixed DDQ. For example, given DDQ *=* 0.8, the average intrusion distance are 2.1846 m, 2.0688 m, 0.8359 m for ACD, ECD, BOAD in Figure 7a when *r* = 5 m. In contrast, when *r* = 15 m, the average intrusion distance are 5.977 m, 4.6228 m, 2.8487 m for ACD, ECD, BOAD, respectively. This mainly illustrates the two issues, namely, ECD and BOAD effectively improved detection quality, and second, in the same DDQ request, the detection performance would be perceived impact of the sensing radius and the number of nodes. We will analyze the effects of network parameters in Section 5.3.

In intrusion detection applications, the intruder may appear in a location with the lowest detection probability or the longest intrusion distance. The results in this section indicate that for a given applications, we can make appropriate choices among the two strategies above according to different application. For example, in applications of border or perimeter surveillance against hostile elements such as embassies, and factories, BOAD has better surveillance performance. In precision agriculture, fire monitoring applications, the ECD can be up to the task.

### 5.3. Effects of Network Parameters

In order to verify the validity of our theoretical results, we investigated the design parameters by performing extensive simulations. Apparently, there are three factors influencing the desired deployment quality: the monitored region, the number of sensor nodes and the sensing range. We assume a monitored region 100 m × 100 m, and the analytical and simulation results are compared by varying the number of deployed sensor nodes and the sensing radius.

First, we examine the impact of the number of deployed sensor nodes with different sensing range on the desired deployment quality of the three strategies.

**Figure 9.**
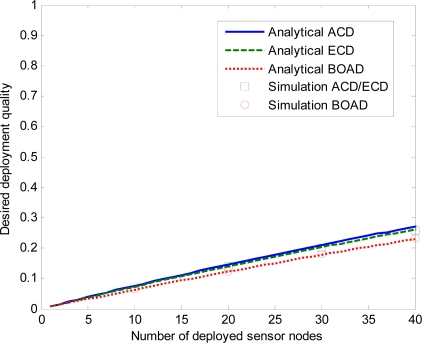
Effects of number of deployed sensor nodes on the DDQ for *r* = 5 m.

**Figure 10.**
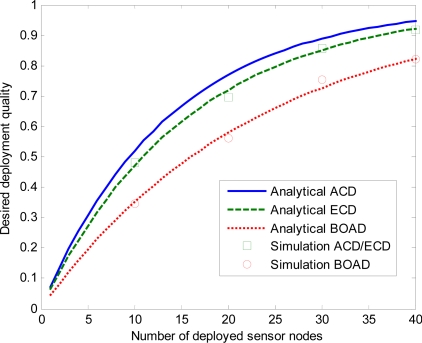
Effects of number of deployed sensor nodes on the DDQ for *r* = 15 m.

**Figure 11.**
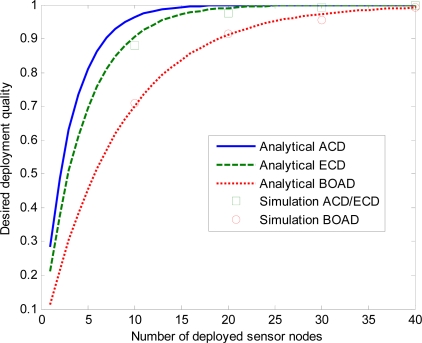
Effects of number of deployed sensor nodes on the DDQ for *r* = 30 m.

Figures 9, 10 and 11 depict desired deployment quality as a function of the number of sensor nodes when sensing range *r* = 5, 15, 30 m. ACD and ECD are under the same monitored region, so the simulation results of these two strategies are the same when same number of sensor nodes are deployed. We denote the simulation ACD/ECD as the simulation results of ACD or ECD. Note that the simulation results match the analytical curves well, which validates the correctness of our derivations. As expected, desired deployment quality increases as *n* increases for the three strategies. From Figure 9, we observe that, both analysis and estimate values of ECD and BOAD strategies are close to the simulation results. The analysis and simulation of ACD is slightly discrepancy. That is to say, for smaller values of *r*, analytical and simulation values do not deviate significantly. This is because when *l* ≫ *r*, *m* ≫ *r*, the ACD can evenly estimate the deployment quality of a sensor network. However, with the *r* becoming larger, the variation increases, as shown in Figures 10 and 11.

Figure 10 and Figure 11 reveal that ECD and BOAD consistent with the simulated results while ACD has a significant error when *r* = 15, 30 m. As *r* increases, the discrepancy gets larger. For example, when *n* = 10, the discrepancy of simulation and analytical result of ACD is 0.0037 when *r* = 5. The discrepancy of simulation and analytical results of ECD and BOAD are 0.0006 and 0.0026, respectively. On the contrary, the discrepancies are 0.0403 and 0.0842 when *r* = 15, 30 m for ACD. The discrepancies are 0.0083 and 0.0264 when *r* = 15, 30 m for ECD and BOAD, respectively.

**Figure 12.**
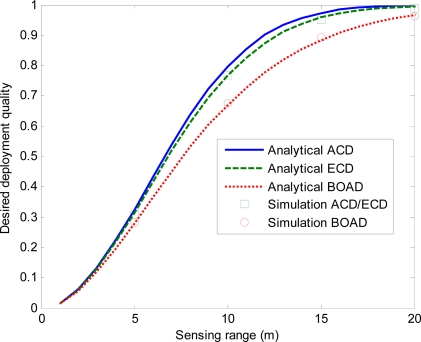
Effects of sensing range on the DDQ for *n* = 50.

In fact, we found that ACD match for the ECD when the radius is small compared to the length or width of the rectangle. Figure 12 shows the results of varying *r* from 0 to 20 and fixed *n* = 50.

From Figure 12, we can see that in the three deployment strategies, the desired deployment quality increases with the extension of sensor’s sensing range. It is because the increase of sensing range improves the network coverage, that it turn improves the deployment quality.

From Figures 9–12, we observe that, BOAD achieves a lower desired deployment quality compared to ECD and ACD under the same deployed number of sensor nodes. This is because some sensor nodes should be deployed outside the monitored field to meet the desired deployment quality. In addition, we can observe that the desired deployment quality is infinitely close to 1 when the number of sensor nodes or the sensing radius is larger than the certain threshold.

The simulation results can be summarized as follows:

1. Both BOAD and ECD can efficiently alleviate the coverage overestimation in terms of the desired deployment quality, which can ensure the surveillance quality.
2. Both BOAD and ECD reduce the average intrusion distance compared to ACD in intrusion detection applications. Furthermore, BOAD, which uses a boundary assistant region, has the best performance in terms of the average intrusion distance when the invasion of intruder is from the boundary of a monitored region. ECD has the best performance in terms of the average intrusion distance when the invasion of intruder is from the inside of monitored region.
3. Achieved desired deployment quality increases when the number of sensor nodes or radius increases. BOAD achieves the lower desired deployment quality compared to ECD under the same number of sensor nodes and sensing radius, the analysis and simulations have slight discrepancy which efficiently alleviate coverage overestimation.

## 6. Practical Discussion

So far, we have analyzed the lower bound number of sensors deployed to achieve a desired deployment quality. In this section, we address two practical issues with random deployment strategies. We first discuss how to apply the derivations in Section 4 to a sensing field of a more general shape and the sensing model to real applications.

### 6.1. General Monitored Region

We first remark that the validity of the derivations of the deployment strategies is not limited to any particular shape of the monitored region. The derivations in Section 4.1 and 4.2 assume that the monitored region is rectangular. However, the methods and the derivations can be extended to the case where the monitored field is of arbitrary convex shape. Assume that the shape of the brim is not too rugged, from Section 4.1, it is apparently introduced boundary effects when 0 ≤ *d*(*i*) < *r*. When *d*(*i*) = 0, the node *i* sits on the boundary and the corresponding coverage area is approximately a half circle, *i.e.*, *Ef*(*d*(*i*)) ≈ *πr*^2^*/2*. Similarly, when *d*(*i*) = *r*, the coverage area is a circle tangent on the boundary, *i.e.*, *Ef*(*d*(*i*)) ≈ *πr*^2^. When 0 < *d*(*i*) < *r*, for analytical tractability we can introduce a linear interpolation to *Ef*(*d*(*i*)) that simply scales linearly in *d*(*i/2*) [29]:

(27) $$\mathrm{Ef}(d(i))=\{\begin{array}{ll} {\pi r}^{2} & \text{if}\;d(i)\geq r\\ \pi r(r+d(i))/2 & \text{if}\;0\leq d(i)\leq r \end{array}$$

Using Equation (27), for the given area and perimeter of the monitored region, we can make approximation accurately about the expected coverage area of nodes according with the sensing radius *r*. Therefore, the lower bound on the number of deployed sensor nodes can derive approximately to fulfill the desired deployment quality.

On the other hand, concerning the boundary assistant deployment strategy discussed in Section 4.2, we can easily extend to arbitrary convex monitored region by using parallel convex sets [30]. By doing so, we give following Lemma 4.

**Lemma 4.** Let *K*_Ω_ be an area of the monitored region with parameter *L*_Ω_ and let sensors with sensing radius *r* randomly deployment, when using boundary assistant deployment strategy, the area of the deployment region *D_r_* and *L_r_* of the BOAD strategy is:

(28) $$\begin{array}{c} D_{r}=K_{\Omega }+rL_{\Omega }+{\pi r}^{2}\\ L_{r}=L_{\Omega }+2\pi r \end{array}$$

### 6.2. Probabilistic Sensing Model

This study assumes that the sensing models of deployed sensor nodes are deterministic and the monitored region is rectangular. In practice, due to the randomness in sensing, ambient noise, interference and obstacles in monitored region, probabilistic models describe a sensor’s sensing ability more accurately.

In wireless sensor networks, the sensing capability is mandatory for monitoring applications that immediately response to detect the event. With unit disk sensing model analyzed in Section 2, we can derive the minimum number of sensors to achieve the required deployment quality. However, the sensing capabilities are affected by the distance between the sensor and the measuring point [12,15,31] and environmental factors in real-world applications [13]. As observed in [13], the general sensing model [12,15,31] cannot well model the shadowing effects on the sensing signal propagation path. In this section, we use the log-normal shadowing model proposed by [13] to discuss our deployment strategies.

To sense an event in monitored region, the sensor nodes should sense the emitted signal from the sensing area. Considering the nodes sensing range *r*, let *S_target_* denote the signal power emitted by a target. The received signal by sensor *i* expressed as *S_rev_*(*r*). We have:

(29) $$S_{\mathrm{rev}}\;(r)=S_{\mathrm{target}}-\mathrm{PL}(d_{0})-10\beta \log_{10}\;(d_{i}/d_{0})+\chi_{\sigma }$$

where *d_i_* denotes the distance between the target and the sensor node; *PL*(*d_0_*) (in decibel units) is the average propagation loss at a reference distance *d_0_*; *β* denotes the path (signal power decay factor) loss exponent which indicates the decreasing rate of signal strength in an intrusion detection environment, and *χ_σ_* is a shadowing sample which is assumed to be Gaussian distributed, with zero mean and standard deviation *σ*.

In shadowed environments, the sensing area of a sensor is not a disk, and the sensing radius is not *r*. The parameters *PL*(*d_0_*), *β* and *χ_σ_* are environment dependent, and the sensing radius of a sensor node is affected by the parameters. By calibrating the propagation model, the average sensing radius *r_ave_* can get for propagation environment *i*:

(30) $$r_{\mathrm{ave}}=d_{0}\times 10^{[S_{\text{target}}-S_{\mathrm{sen}}-{\mathrm{PL}}_{i}(d_{0})]/10\beta }$$

where *S_sen_* is the received signal power without shadowing environments. For a detail derivation, please see ref. [13].

Therefore, given the *r_ave_*, we can easily extend the probabilistic sensing model to the deployment strategies ECD and BOAD.

## 7. Conclusions and Future Work

Balancing the deployment quality and deployment cost is a challenging task in sensor networks under random deployment. Aiming at reducing coverage overestimation, we proposed two node deployment strategies in wireless sensor networks. Specifically, we studied network coverage by randomly deployed sensor nodes, and obtained the lower-bound of number of sensor nodes which can accurately meet the deployment requirement. The performance study of the deployment strategies shows the new strategies have significant advantages to the area coverage deployment. We also evaluate the performance in an intrusion detection application. Boundary assistant deployment strategy showed the best performance when the invasion of intruder comes from the boundary of monitored region. On the other hand, expected-area coverage deployment strategy has the best performance when the invasion of intruder happens from inside of a monitored region. Moreover, the theorems that we have derived characterize the interactions among network parameters. The results obtained in this paper will provide important guidelines for the random deployment of typical application of wireless sensor networks and our analysis can help to plan a sensor network meeting deployment quality requirements at a low budget cost.

Network connectivity, in addition, reflects how well the sensor nodes communicate with each other in reporting detected events or the sensed data to the sink node. For our future work, we plan to study the connectivity under two deployment strategies and give a detail analysis and simulation when extended to general monitored region and probabilistic sensing model.

## Figures and Tables

**Table 1.** Notation and definition.

Notation	Definition
Ω	the monitored region
*D*	the deployed region
*B*	the boundary assistant region
*n*	the number of deployed sensor nodes
*n^k^*	the lower bound number of deployed sensor nodes for strategy *k*
*r_i_*	the sensing radius of sensor *i*
*A*(*x*)	the circular area centered at given point *x* with radius *r*
*d*(*i*)	the distance to the boundary of monitored region of sensor *i*
*Ef*(*d*(*i*))	the effective coverage area of sensor *i*
*E_r_*[*j*]	the expected area of *sub-region j*
Ω*_r_*(*j*)	the sub-region *j* of monitored region

**Figure 13.**
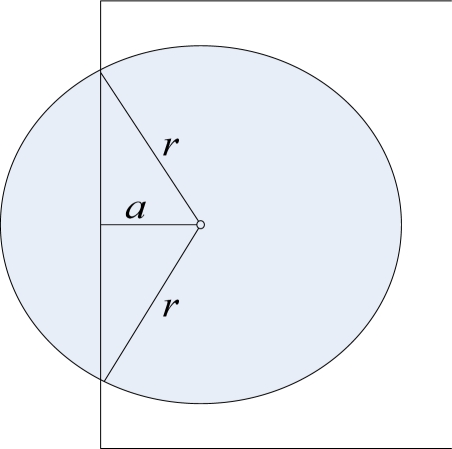
A sensor node located in sub-region Ω*_r_*(1).

**Figure 14.**
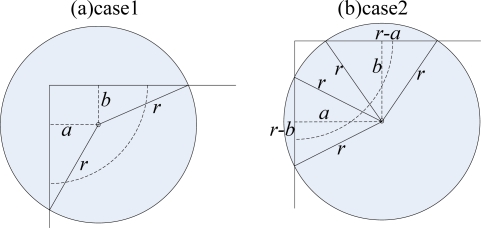
Two cases of a sensor’s location in sub-region Ω*_r_*(2).

## Appendix

In order to compute *E_r_*[1] and *E_r_*[2], we need the following results from [32].

### Computing *E_r_*[1]

Let *a* denote the distance from a node located in Ω*_r_*(1) to the border of Ω. For a given *a*, the overlapped area of the sensor’s sensory region and the monitored region is (see Figure 13):

(31) $$A_{\Omega_{r}(1)}(a)=a\sqrt{r^{2}-a^{2}}+(\pi -\text{arccos}(a/r))r^{2}$$

Since 0 ≤ *a* ≤ *r*, let *E_r_*[1] denote is the expected value of *A*_Ω_*r*_(1)_(*a*), we have:

(32) $$E_{r}\;[1]=\frac{1}{r}\int_{0}^{r}A_{\Omega_{r}\;(1)}\;(a)\mathrm{da}=\frac{1}{r}\int_{0}^{r}(a\sqrt{r^{2}-a^{2}}+(\pi -\text{arccos}(a/r))r^{2})\mathrm{da}$$

Hence, after few steps of mathematical simplification, we derived:

(33) $$E_{r}\;[1]=(\pi -\frac{2}{3})r^{2}$$

### Computing *E_r_*[2]

Let the distance from a node located in Ω*_r_*(2) to the two borders of rectangle be *a* and *b*, respectively (see Figure 14). Depending on the location of the sensor node, two cases are possible.

Case 1. The distance to the corner is less than *r* (Figure 14a).
Case 2. The distance to the corner is larger than or equal to *r* (Figure 14b).

Let *E*_*r*1_[2] and *E*_*r*2_[2] denote the expected coverage in Case 1 and Case 2, respectively. We have

(34) $$E_{r}\;[2]=\frac{\frac{1}{4{\pi r}^{2}}}{r^{2}}E_{r1}\;[2]+(1-\frac{\frac{1}{4{\pi r}^{2}}}{r^{2}})E_{r2}\;[2]$$

We then compute *E*_*r*1_[2].

Let *A*_Ω_*r*1_(2)_(*a, b*) denote the overlapped area of the node’s sensory region and the deployment region in Case 1. By geometry we have:

(35) $$A_{\Omega_{r1}\;(2)}\;(a,b)=\mathrm{ab}+\frac{a\sqrt{r^{2}-a^{2}}}{2}+\frac{b\sqrt{r^{2}-b^{2}}}{2}+{\pi r}^{2}\left(1-\frac{\text{arccos}(\frac{a}{r})+\text{arccos}(\frac{b}{r})+\frac{\pi }{2}}{2\pi }\right)$$

Then, we have:

(36) $$E_{r1}\;[2]=\frac{1}{\frac{1}{4}{\pi r}^{2}}\int_{0}^{r}\int_{0}^{\sqrt{r^{2}-a^{2}}}A_{\Omega_{r1}\;(2)}\;(a,b)\mathrm{dadb}$$

By simplifying, we get:

(37) $$E_{r1}\;[2]=\frac{(\pi^{2}+1)r^{2}}{2\pi }$$

Compute *E*_*r*2_[2].

Let *A*_Ω_*r*2_(2)_(*a, b*) denote the overlapped area of the node’s sensory region and the deployment region in Case 2. We have:

(38) $$A_{\Omega_{r2}\;(2)}\;(a,b)=a\sqrt{r^{2}-a^{2}}+b\sqrt{r^{2}-b^{2}}+{\pi r}^{2}\left(1-\frac{\text{arccos}(\frac{a}{r})+\text{arccos}(\frac{b}{r})}{\pi }\right)$$

Similar technique used in computing *E*_*r*1_[2] can be used here. It turns out that:

(39) $$E_{r2}\;[2]=\frac{4r^{2}(\pi -\frac{4}{3}-\frac{\pi^{2}}{8})}{4-\pi }$$

Since $E_{r}\;[2]=\frac{\frac{1}{4{\pi r}^{2}}}{r^{2}}E_{r1}\;[2]+(1-\frac{\frac{1}{4{\pi r}^{2}}}{r^{2}})E_{r2}\;[2]$

We have:

(40) $$E_{r}\;[2]=(\pi -\frac{29}{24})r^{2}$$
